# From Cognitive Load Theory to Collaborative Cognitive Load Theory

**DOI:** 10.1007/s11412-018-9277-y

**Published:** 2018-04-25

**Authors:** Paul A. Kirschner, John Sweller, Femke Kirschner, Jimmy Zambrano R.

**Affiliations:** 1 Open University of the Netherlands, Valkenburgerweg 177, 6419AT Heerlen, The Netherlands; 2 University of Oulu, Oulu, Finland; 3 University of New South Wales, Sydney, Australia; 4 Utrecht University, Utrecht, The Netherlands; 5 Universidad de Los Hemisferios, Quito, Ecuador; 6 Instituto Tecnológico Superior Rumiñahui, Sangolquí, Pichincha Ecuador

**Keywords:** Cognitive load, Collaborative learning, Collaborative cognitive load theory, Transactive activities, Computer supported collaborative learning

## Abstract

Cognitive load theory has traditionally been associated with individual learning. Based on evolutionary educational psychology and our knowledge of human cognition, particularly the relations between working memory and long-term memory, the theory has been used to generate a variety of instructional effects. Though these instructional effects also influence the efficiency and effectiveness of collaborative learning, be it computer supported or face-to-face, they are often not considered either when designing collaborative learning situations/environments or researching collaborative learning. One reason for this omission is that cognitive load theory has only sporadically concerned itself with certain particulars of collaborative learning such as the concept of a collective working memory when collaborating along with issues associated with transactive activities and their concomitant costs which are inherent to collaboration. We illustrate how and why cognitive load theory, by adding these concepts, can throw light on collaborative learning and generate principles specific to the design and study of collaborative learning.

This article discusses an expansion of cognitive load theory from individual learning to collaborative learning. As such, it attempts to help solve the conundrum of why collaborative learning in general, and computer-supported collaborative learning (CSCL) specifically, sometimes works while at other times fails. At best, one can say that its purported benefits are not always consistent (Kester and Paas 2005; Kirschner et al. 2009a; Slavin 2014). This variation could be because research has paid little attention to the human cognitive architecture that underlies group processes, the prior group experience, and the information distribution among collaborators. While the use of cognitive load theory has led to specific instructional design principles based on, for example, split-attention and redundancy effects, instructional tools and scaffolds (e.g., process worksheets, worked examples), and even instructional design methods (e.g., Four Component Instructional Design, Van Merriënboer, 1997; Ten Steps to Complex Learning, Van Merriënboer & Kirschner, 2018), such design principles have not been identified for collaborative learning. Those working and researching in the field often do not make use of cognitive load theory, neither for designing collaborative learning instructional situations and environments nor in the research being carried out on CSCL.

The current expansion applies to collaborative learning in all its forms and flavours, regardless of whether it is collocated/contiguous where group^1^ members study and learn at the same time and in the same place or whether learners are spread across the globe working synchronously or asynchronously with the support of computers and computer networks (i.e., CSCL). The article begins with a discussion of evolutionary psychology, human cognitive architecture, and instructional design and relates these to collaborative learning. It then follows with a discussion of the advantages of learning collaboratively and the transactive activities involved in collaboration. The article concludes with a number of principles relating to the use of collaborative learning in light of cognitive load along with its use and meaning for CSCL research.

## Evolutionary Psychology, Human Cognitive Architecture and Instructional Design

The instructional design recommendations of cognitive load theory are based on a version of human cognitive architecture that in turn can be derived from evolutionary psychology (Sweller 2016a, 2016b; Sweller et al. 2011a, 2011b). We will indicate the close theoretical relations that can be established between evolutionary psychology, cognitive architecture and instructional design. These theoretical relations provide the core of cognitive load theory.

### Evolutionary Educational Psychology

The distinction between biologically primary and biologically secondary knowledge^2^ described by Geary is an instructionally important categorisation scheme (Geary 2008, 2012; Geary and Berch 2016). *Biologically primary knowledge* is knowledge we, as a species, have specifically evolved to acquire over many generations. Primary skills, such as learning general problem solving strategies, imitation, recognising faces, communication through listening and speaking a native language, and social relations including our ability to communicate with each other, are modular with each skill likely to have evolved during different evolutionary epochs. We can acquire primary knowledge, easily, unconsciously, and without explicit instruction merely by membership in a group. Generally, because primary skills are acquired effortlessly, they do not need to be formally taught. Most generic-cognitive skills such as problem-solving, planning, or generalising are biologically primary (Sweller 2015; Tricot and Sweller 2014). Communicating by speaking and joint attention is a generic-cognitive skill (Callaghan et al. 2011; Tomasello & Rakoczy, 2003).

The ability to acquire vast aspects of the culture we grow up in is biologically primary. Nevertheless, in most cultures, there are many concepts and procedures that we have not specifically evolved to acquire such as reading, doing mathematics, working with a computer, or searching the internet. Those *biologically secondary skills* are acquired consciously, often requiring considerable effort. Unlike primary knowledge and skills, explicit instruction is important when dealing with secondary knowledge and skills (Kirschner et al. 2006; Sweller et al. 2007). Without explicit instruction, this knowledge acquisition is likely to be severely compromised.

Unlike the generic-cognitive skills that tend to be biologically primary, biologically secondary skills tend to be *domain-specific* (Sweller 2015; Tricot and Sweller 2014). Examples of biologically secondary skills include almost everything that is taught in education and training institutions. The distinction between primary, generic-cognitive knowledge and secondary, domain-specific knowledge explains why information tends to be acquired differently outside as opposed to inside educational contexts. We use primary knowledge to leverage acquiring secondary knowledge (Paas and Sweller 2012). For example, to learn geometry in a conventional class or using computer-supported material requires primary skills such as visual recognition, join attention, and schemas about space, time and sequence (Casasanto et al. 2010; Núñez and Cooperrider 2013; Siegel and White 1975), to name a few.

In this way, the theoretical machinery of evolutionary educational psychology can be used to suggest that the primary, generic-cognitive knowledge associated with collaborative learning may, under some circumstances, improve the acquisition of the biologically secondary, domain-specific knowledge that is taught.

### Human Cognitive Architecture

The manner in which biologically secondary knowledge is processed by the human cognitive system is analogous to the way in which evolution by natural selection processes information. Both are examples of natural information processing systems (Sweller and Sweller 2006) which can be described using five principles summarised in Table 1.

**Table 1:** Natural information processing system principles

Principle	Function
Information store	Store information in long-term memory for indefinite periods
Borrowing and reorganising	Permit the rapid building of a long-term memory store by borrowing information from another person’s long-term memory
Randomness as genesis	Create novel ideas
Narrow limits of change	Use limited working memory to process novel information
Environmental organising and linking	Use environmental signals to transfer organised information from long-term memory to working memory in order to effect appropriate action

The *information store principle* indicates that in order to function, natural information processing systems require an enormous store of information. Long-term memory provides that store for primary and secondary knowledge in the case of human cognition. The finding that skilled performance in any complex area requires the memorisation of tens of thousands of problem states and the best moves for each state (De Groot and Gobet 1996; Egan and Schwartz 1979; Jeffries et al. 1981; Sweller and Cooper 1985) provided evidence for the importance of long-term memory to general cognition. The ability to store information in long-term memory is a biologically primary skill that does not need to be taught.

The second principle, the *borrowing and reorganising principle*, suggests that most of the information acquired by and stored in long-term memory is borrowed from the long-term memories of other people. We imitate others, listen to what they say and read what they write. Once information is acquired from others, it is reorganised by us using information previously stored in our long-term memory (Bartlett 1932).

For the purpose of this article, there are two aspects of this principle that need to be noted. First, borrowing and reorganising knowledge from others does not need to be taught because it is biologically primary. We are one of the few species that has evolved to obtain information from others (Brownell et al. 2006). Second, collaborative learning makes use of the borrowing and reorganising principle and is one of the justifications for hypothesising that collaboration can be effective for learning. During collaboration, we can obtain important information from others that may be difficult to obtain by other means. Of course, while most explicit instruction, both oral and written, also makes use of this principle, collaboration differs from non-collaborative instructional methods because there may be a greater emphasis on the reorganising aspect of this principle.

The *randomness as genesis principle* explains how information is first generated. If we are unable to obtain needed information from others, we need to use our primary skills to generate information ourselves during problem solving. In the absence of sources that allow us to borrow required information, we must randomly generate problem-solving moves and test them for their effectiveness. Again, this procedure is biologically primary and does not need to be formally taught. We have evolved to use general problem solving strategies and to generate moves randomly and test them for effectiveness.

The fact that the randomness as genesis principle is used in important activities such as research does not justify its use when information can readily be borrowed from others. Problem solving is only useful when we do not have alternative access to problem solutions. Under appropriate circumstances, collaborative learning can provide that access by increasing the range of information available to us.

The randomness as genesis principle has functional implications for the cognitive system, leading to the the fourth principle, namely the *narrow limits of change principle*. In order to avoid combinatorial overload and explosions, we need a structure that limits the number of elements of information that we can consider at one time. Those limits are imposed by our working memory that is severely limited in both capacity (Miller 1956) and duration (Peterson and Peterson 1959). It needs to be noted that those limits only apply to novel information and not to familiar information retrieved from long-term memory, as will be discussed under the next principle. It also needs to be noted that collaborative learning may ameliorate some of the limitations of working memory (F. Kirschner et al. 2011) and especially that of asynchronous CSCL where written text is often used which may lead to cognitive offloading (Hmelo-Silver, 2002; Suthers 2006). By having multiple working memories working together on the same task, the effective capacity of the multiple working memories may be increased due to a *collective working memory effect* that is discussed in more detail below.

The *environmental organising and linking principle* is the fifth principle and provides a justification of the preceding principles. Signals from the environment trigger the transfer of appropriate information from long-term memory to working memory. That information can then be used to generate action appropriate to the environment. While working memory is limited when dealing with novel information, it has no known limits when dealing with organised information from the information store of long-term memory. Based on this principle, we are transformed by our ability to marshal large amounts of information transferred from long-term memory to working memory. These large amounts of information from long-term memory can be held in working memory indefinitely giving us an ability to carry out actions that otherwise we could not consider. Accordingly, one of the primary aims of instruction is to help learners to accumulate the large stores of secondary knowledge and skills in long-term memory for later use. Collaborative learning aims to facilitate that procedure by increasing our ability to collectively process novel information.

In considering the advances of the evolutionary perspective and the application of the principles of human architecture to collaborative learning leads to a sub-principle, the *mutual cognitive interdependence principle* (Tomasello and Gonzalez-Cabrera 2017; Tomasello, Melis, Tennie, Wyman, & Herrmann, 2012). This sub-principle acts as a subsidiary of the borrowing and re-organising principle by detailing how cognitive systems (i.e., inter-cognitive processes) acquire information between them. Systems develop, process, create, acquire, and share knowledge in mutual openness and collaboration with other systems. The knowledge in long-term memory that has been acquired by students consists of elaborations and structures intrinsically related to the type of relationship with others (i.e., instructors, other learners) and the means by which they carry out their cognitive transaction activities (i.e., face-to-face, mediated by computers). Individuals depend on an instructor’s explicit guidance and appropriate interactions with others as part of a group, but also on appropriate instructional environments of collaboration with other learners. This principle presupposes a relative openness between cognitive systems (Scheler 1994) and pays attention to the intrinsic transactive processes that allow cognitive exchange between them (Zambrano et al. 2017b). In addition, it takes into account the relationship between the system(s) and the environment without reducing them to the cognitive components of an individual system. Consequently, the evolution of human cognitive architecture depends on the mutual and simultaneous relationship between the components of an information-processing system, between systems, and between the systems and their environment.

### Instructional Design

*Cognitive load theory* has used this cognitive architecture to devise cognitively effective and efficient instructional procedures. Cognitive load refers to the total working memory resources required to carry out a learning task. It assumes that human memory can be divided into two basic forms, working memory and long-term memory, that the information that is stored in long-term memory takes the form of schemas, and that the processing of new information requires mental effort resulting in cognitive load on working memory which affects learning outcomes (Sweller, 1998).

When presented with novel information, there are two additive sources of cognitive load imposed on working memory (Sweller 2010): intrinsic and extraneous load. In addition, germane cognitive load, defined as the working memory resources devoted to dealing with intrinsic cognitive load, is frequently discussed but it is closely related to intrinsic cognitive load. Kalyuga (2011, p. 1), for example posited that:

[I]n its traditional treatment, germane load is essentially indistinguishable from intrinsic load, and therefore this concept may be redundant … the dual intrinsic/extraneous framework is sufficient and non-redundant and makes boundaries of the theory transparent. The idea of germane load might have an independent role within this framework if (as recently suggested by John Sweller) it is redefined as referring to the actual working memory resources devoted to dealing with intrinsic rather than extraneous load.

As such, germane load is not treated as an additive source of load here.

*Intrinsic cognitive load* deals with the inherent complexity of the information that needs to be processed. Complexity, in turn, is defined in terms of element interactivity. Consider a learning task given by a teacher such as learning the translation of a list of 50 words from one language to another (i.e., word-pairs) within a certain period of time (e.g., 60 min). Despite the difficulty of learning the words, it is not a complex task because each word-pair can be learned independently of every other word-pair. Learning that *chat* is the French word for cat can be learned without reference to the fact that *chien* is the French word for dog. The two word-pairs do not interact. For this task, element interactivity and intrinsic cognitive load are low because working memory does not have to process more than one or two word-pairs simultaneously. Of course this intrinsic load will be influenced to a certain extent by the learner’s prior knowledge, for example if the learner knows a different Indo-European or more importantly Romance language than French (e.g., Spanish, Italian, Portugese, etc.) the task could be, intrinsically, less complex and also less difficult while for a learner without knowledge of either English or French (e.g. someone who speaks only Slavic or Afroasiatic languages) the task is intrinsically more complex and more difficult. In contrast, using those same words to write a few simple sentences requires far fewer elements but the elements interact with each other. All of the words in the sentence have relations with other words (e.g., gender, gender-related articles, plurals, verb conjugation, etc.) and thus must be considered as a whole unit in working memory when learning to carry out this task. We are often unable to make any change to any of the parts of the sentence without affecting other elements and so element interactivity and intrinsic cognitive load are high.

Element interactivity is affected by both the nature of the task (as indicated) and by levels of learner expertise. In the above example, learners who are competent in a language have stored the grammatical relations between words in long-term memory. According to the *environmental organising and linking principle*, that stored knowledge can be transferred to working memory as a single entity. In other words, the interacting elements are incorporated in the stored knowledge and so an entire problem such as writing The translation of the sentence *“cats and dogs are both pets”* into French “*les chats et les chiens sont tous deux des animaux domestiques”* constitute a single larger and more complex chunk (Egan and Schwartz 1979) or encapsulated element (Boshuizen & Schmidt, 1992). The individual elements are incorporated in that chunk and so for an expert in the language, element interactivity is low. For a novice, element interactivity for this collection of words may be very high. A change in either the nature of the task or the expertise of the learner results in a change in element interactivity which otherwise, remains constant.

Element interactivity also determines the level of *extraneous cognitive load*. This form of working memory load refers to the load imposed by information elements unrelated to the learning task such as the way the information or the task is presented (Chen et al. 2016). These elements can be produced by instructional procedures and so it is under the control of instructors and can be varied by using different instructional procedures. Cognitive load theory has developed a wide range of instructional procedures designed to reduce extraneous cognitive load (Sweller 2011). Another example is the worked example effect which occurs when problem-solving skill is enhanced more by studying worked examples rather than solving the equivalent problems. The effect occurs because element interactivity is reduced by studying worked examples in comparison to problem solving. During problem solving, learners must search for appropriate moves using the *randomness as genesis principle*. In the case of an algebra problem such as *(a + b)/c = d, solve for a*, all of the elements of the problem statement must be considered in an interconnected way along with the consequences of the series of possible moves at each choice point. Element interactivity may result in a working memory load far above working memory limits. In contrast, studying a worked example demonstrates a use of the *borrowing and reorganising principle*. When studying a worked example, each step can be considered without concurrently considering alternative moves because an appropriate move has been provided. Element interactivity and the extraneous load on working memory are reduced by the use of worked examples.

### Relation to Collaborative Learning

Collaborative learning occurs when two or more students actively contribute to the attainment of a mutual learning goal and try to share the effort required to reach this goal, either face-to-face or supported by a computer (Teasley and Roschelle 1993). This activity is most often initiated by the posing of a learning task or problem by the instructor. The task may be well-defined (i.e., a task with specific goals, clearly defined solution paths, and clear expected solutions), ill-defined (i.e., a task with no clear goals, solution paths, or expected solutions) or even wicked (i.e., a task with incomplete, contradictory, and/or changing requirements that are often difficult to recognize; Rittel and Webber 1984). Many researchers of CSCL have emphasised the use of learning in groups for all three types of tasks/problems (Baghaei et al. 2007; Le et al. 2013; Scheuer et al. 2010; Strijbos, Kirschner, & Martens, 2003; Suthers 2003). The use of cognitive load theory for these different types of tasks has also been well recorded, for example Van Merriënboer and Sweller (2010), Rourke and Sweller (2009), and Sweller et al. (2011a, 2011b).

Although in the short run, collaborative learning results in group members trying to successfully perform a certain learning task or solve a specific problem together, in the long run, as an instructional method, it is very important that all members of the group develop effective experience working together (i.e., domain-generalised group knowledge, (Kalyuga 2013)) that facilitates every member in acquiring domain-specific knowledge from this combined effort.

The use of collaborative learning has implications for extraneous cognitive load. Let us assume students are learning to solve a particular class of geometry problems. Depending on the extent to which the elements interact, there is an intrinsic cognitive load associated with that task irrespective of how it is taught. In addition, we need to choose whether to have the students learn this material individually or collaboratively. Both instructional procedures have levels of element interactivity associated with them that are independent of the intrinsic cognitive load. Indeed, since ‘individual’ learning and ‘collaborative learning’ are extremely broad umbrella terms, the levels of element interactivity associated with both depend on the particular version of individual or collaborative learning we use. Our aim is to reduce the element interactivity associated with extraneous cognitive load and optimise the elements associated with instrinsic cognitive load by changing the instructional technique we use. If the element interactivity associated with collaborative learning (i.e., for the individual group member) is less than the element interactivity associated with individual learning, then extraneous cognitive load is reduced by using collaboration.

There are theoretical grounds for hypothesising that the use of collaborative learning can reduce element interactivity and its concomitant cognitive load. According to the *mutual cognitive interdependence principle*, appropriate collaborative learning introduces a *collective working memory* (F. Kirschner et al. 2011) that otherwise does not exist. This collective working memory is part of a collective working space that is created by communicating and coordinating (relevant) knowledge held by each individual group member. Through communication and the resulting socio-cognitive processes within the group, a collective knowledge structure, or mutually shared cognition, consisting of shared mental models is formed. Research shows that these collective knowledge structures are conditional for the effectiveness of collaboration (Van den Bossche et al. 2006). The concept of a collective working memory is strongly linked to the theory of group cognition (Stahl 2014) which considers a larger unit of analysis than the individual mind as a producer of cognitive activities such as complex problem solving. However, the collective working memory concept has an important focus on the learning of individuals in the group. Under individual learning, all interacting elements must be processed in a single working memory of that individual. Under collaborative learning, various interacting elements can be distributed among multiple working memories (i.e., the working memories of the different group members) thus reducing the cognitive load on a single working memory. Those multiple working memories constitute a collective working memory that is larger than a single memory. One could state that for complex tasks/problems, collaboration becomes a scaffold (just like worked examples) for individuals’ knowledge acquisition processes. Collaboration, then, will be effective if it becomes a scaffold in this sense. If it does not, or if it in itself adds too much extraneous load, it will be harmful. The process of creating a collective working memory can be supported by helping the members of a group to exchange knowledge and information. Making learners dependent on each other, either for successfully carrying out and completing a task (i.e., task/goal interdependence) or for exchanging resources (i.e., positive resource interdependence), has been shown to be way of doing this (Johnson et al. 2001; Langfred 2000).

In summary, extraneous – and thus also total - cognitive load is changed because having learners collaborate, in effect, changes the instructional procedure (P. Kirschner et al. 2014). A collective working memory function is also seen in CSCL when learners socially share learning, their resources and regulation as is the case in co-regulated and socially shared regulation of CSCL (Järvelä et al., 2015). During collaborative learning, some information comes from collaborators rather than other sources and that information is likely to become available exactly when it is needed resulting in a decreased load and increased learning.

### Collaborative learning and evolutionary categories of knowledge

We can assume humans have evolved to work together, with the existence of language providing strong evidence. Collaboration provides a major purpose for the evolutionary development of language (Tomasello 2008; Tomasello & Rakoczy, 2003). If we have evolved to collaborate, then the act of collaboration is biologically primary. Nevertheless, while we may have evolved to collaborate, it does not necessarily follow that we collaborate effectively and efficiently while acquiring biologically secondary information under all circumstances. A failure to collaborate appropriately may be even more prevalent in CSCL where some affordances/conventions of contiguous collaboration do not apply (e.g., Kirschner 2002a; Jeong and Hmelo-Silver 2016) and where others (e.g., deixis, body language, facial expressions) are often not available (Dwyer & Suthers 2006). Acquiring biologically secondary information during collaboration requires learners to collaborate on a specific secondary task including obtaining the necessary support and guidance to collaborate appropriately. While collaborating is biologically primary, the manner in which we collaborate may differ when, for example, we collaborate to solve a mathematical problem as opposed to write prose or design an artefact, or when we collaborate face-to-face in a project room setting or do the same in a text-based CSCL setting. We may need to learn the differing collaborative techniques for each activity and each setting. It is possible that under some circumstances, collaboration facilitates the learning of biologically secondary information while under other circumstances it interferes with that learning.

Consider two conditions under which collaboration may occur. First, individuals may collaborate because the learning task is highly complex. However, the knowledge held by different people is asymmetric (i.e., each learner may possess some of the necessary information, but not other information that is possessed by other people). In this situation, the task requires collaboration considering the different levels of knowledge and expertise. The goal is learning while carrying out a complex task. However, if the prior knowledge differences have not been recognized before carrying out the task and the members have not had previous experience working together, their learning will be negatively affected (Zambrano et al. 2017b; Zhang et al. 2016). Collaborators will experience extraneous cognitive load due to task-unrelated transactive activities. Some of them may learn incidentally due to primary knowledge, but may not learn as a group.

A second circumstance in which collaboration may occur is when the learning task is highly complex but group members have worked together as a team or they are provided with external collaboration scripts (Fischer et al. 2013). As in the first situation, group members are going to carry out the task. The difference is that they have had experience of how to work together (i.e., how to organize the information, how to distribute the activities among them, how and when to exchange roles according to the type of activity, and so forth), or are explicitly guided by the learning environment as to how to effectively collaborate (e.g., via external scripts, just-in-time support). In other words, collaborators are using their own experience of how to work together or other people’s experience of how to work together so that hey are able to focus their cognitive resources on acquiring relevant knowledge in long-term memory. These collaborators will experience less cognitive load and better knowledge structures due to *task related transactive activities*. A recent meta-analysis provides evidence that CSCL scripts substantially improve learning outcomes for domain-specific knowledge and collaborative skills compared to unstructured CSCL (Vogel et al. 2016).

The above examples show the importance of making well-thought-out choices when it comes to the learning goals of a collaborative task. While in education the goal of learning domain-specific knowledge is often accompanied with the goal of learning how to collaborate, it is important to realise that both require different guidance and support and that what may cause intrinsic load with respect to one goal may produce extraneous load with respect to the other and vice versa. For example, a collaboration script may provide intrinsic load with respect to the learning of a collaboration skill, but attract students’ attention away from a deep processing of the content material being discussed.

From a cognitive load theory perspective, there are conditions under which collaboration may or may not facilitate learning depending on element interactivity and interactions between the *information store principle*, the *borrowing and reorganising principle* and the *narrow limits of change principle*. Collaborative learning is beneficial when the task exceeds individual working memory capacity (under time restrictions) assuming members have not stored relevant prior knowledge structures. Under those circumstances and where individuals have prior experience working together on similar tasks, they can appropriately distribute the elements and cognitive activities of the task at hand and take advantage of their greater capacity and inter-individual communication to acquire better knowledge structures. However, if most or all members already have relevant knowledge structures about the task in their long-term memory, then previous group experience, greater group cognitive capacity, and inter-individual communication are unnecessary. Finally, if groups are composed of advanced students (i.e., more-knowledgeable learners) and are instructed with information already learned, collaboration can even be detrimental as the group members can experience an *expertise reversal effect* that occurs when instructional procedures that are beneficial for novices have negative consequences for more expert learners (Sweller et al. 2011a, 2011b). In sum, what students already know may determine whether collaboration is effective (Retnowati et al. 2017; Zambrano et al. 2017b; Zhang et al. 2016).

Incorporating the *mutual cognitive interdependence principle* into human cognitive architecture used by cognitive load theory provides the basis for the *collective working memory* effect (F. Kirschner et al. 2011; P. Kirschner et al. 2014). This effect suggests that learning in a team is more effective than individual learning if the complexity of the to-be-learned material is so high that it exceeds the limits of each individual learner’s working memory. In this situation, the cognitive load of processing this complex material is shared among the members of a collaborative learning team enabling more effective processing and easier comprehension of the material. In other words, when the complexity of the material which is to be learned and/or the learning task that needs to be carried out is so complex that it exceeds the working memory capacity of the individual learner, the collective working-memory effect will make group learning more effective than individual learning. F. Kirschner et al. (2011) have experimentally confirmed this hypothesis, suggesting that

…for high-complexity tasks, group members would learn in a more efficient way - both in terms of the learning process and outcomes - than individual learners, while for low-complexity tasks, individual learning would be more efficient… This efficiency is affected by the trade-off between the possibility to divide information processing amongst the WMs of the group members (i.e. collective working memory effect) and the associated costs of information communication and action coordination. (p. 621)

Communication and coordination, depending on their content, can be divided into two categories: firstly, general communication and coordination which can be biologically primary, and secondly, school task-specific communication and coordination which is biologically secondary and is based on knowledge of general communication. Biologically primary knowledge will impose little load on working memory (e.g., reading nonverbal communication of team members or making facial expressions in quotidian situations), while biologically secondary knowledge will probably impose a greater load on working memory. Concerning the load on working memory when dealing with collaboration and the channel through which this communication takes place should be taken into account. The more the channel of collaboration mimics a face-to-face interaction, the less of a load collaboration will place on working memory because it relies on biologically primary knowledge we have on how to collaborate with each other. Whether the costs are low or high, both should be taken into account when deciding the effectiveness of collaborative learning as an instructional method. Within the collective working memory effect these costs are refered to as transactive activities, which were introduced above but will be discussed in more detail in the next section.

## Transactive Activities

Transactive activities play a crucial role in the efficiency and effectiveness of collaborative learning. These activities which may occur synchronously or asynchronously (Popov et al. 2017) enable groups to acquire collective knowledge of who the others are and how they can deal with the task (i.e., a collective executive function), the group’s accuracy and willingness to resolve it, and how all members should coordinate what they are doing with each other to accomplish the task together by mediating the acquisition individual and group domain-specific knowledge and the shared, generalised knowledge (Kalyuga 2013; Prichard and Ashleigh 2007). As stated by Popov et al. (2017) “learning is particularly likely to occur when the collaborating students engage in transactive discourse (i.e., critique, challenging of positions and attainment of synthesis via discussion), because this form of discourse gives rise to cognitive activities that stimulate knowledge construction” (p. 426).

It follows that bringing together a group of learners with the relevant knowledge to solve a task is no guarantee that they will work and learn properly (i.e., effectively, efficiently, and without interpersonal problems) both as a group and individually within the group. They must develop a shared mental model and/or a collective scheme of cognitive independence on how to effectively communicate and coordinate their actions so as to share group knowledge, appropriately distribute available task information, and exploit the quality of participation of each group member in the solution of the problem at hand (Hollingshead 2010). To develop collective knowledge, learners should unfold appropriate and efficient transactive activities and be willing to expend resources on collaborative tasks (Fransen et al. 2013; Noroozi et al. 2013; Premo et al. 2017; Prichard and Ashleigh 2007).

### Transactive activities in terms of collaborative learning and cognitive load

Succesful collaborative learning requires communication within a team along with the coordination of collaborative activities. This entails achieving agreement on task-related strategies, dividing tasks between participants, identifying and resolving conflicts, building upon each other’s ideas, achieving consensus, and establishing chronological order of activities (Baker 2002; Erkens et al., 2006; Fransen et al. 2013; Mayordomo and Onrubia 2015; Popov et al. 2017). This communication and coordination brings with it costs to the learners in terms of cognitive load (Ciborra & Olson, 1988; F. Kirschner et al. 2009b; Yamane, 1996). Popov et al. (2017) give a very relevant example with respect to temporally synchronizing communication and coordination activities in CSCL noting that with respect to – among other things - the temporal synchronicity within a team, if the activities of the team members are not aligned or are poorly aligned, the carrying out of the learning task along with the subsequent learning from that task will be negatively influenced.

The concept of transaction costs originated in the field of economics and was used to denote costs other than the monetary price of a good or service, incurred in trading goods or services such as search and information costs (e.g., finding a supplier or price), bargaining and decision costs (e.g., legal and notarial fees, contract negotiation time and expenses), and policing and enforcement costs (e.g., monitoring, policing and/or enforcing what was agreed upon) (North & Thomas, 1973).

A collaborative or cooperative learning environment has analogous transaction costs that can be described as

the costs of setting up, enforcing, and maintaining the reciprocal obligations, or contracts, that keep the members of a team together [and]…represent the “overhead” of the team…linked to the resources (time, skills, etc.) employed to allow a work team to produce more than the sum of its parts (Ciborra & Olson, 1988, p. 95).

In cognitive load theory, communicating and coordinating costs due to collaboration are associated with the specific extra acts that a learner has to carry out when studying, namely communicating with other learners, and coordinating both their own learning and the learning of other team members (Janssen et al. 2010; F. Kirschner et al. 2009b).

Due to the effect of communication and coordination on cognitive load and therefore on the effectiveness of collaborative learning environments, structure and control of communication and coordination of biologically secondary domain-specific knowledge are very important. The beneficial effect of being able to share the cognitive load within a group could be annulled by the costs of communication and coordination (i.e., cognitive load caused by transactive activities) between the group members. These costs may be even more important in CSCL environments where communication and coordination may be hampered by the specific affordances and/or shortcomings in those affordances of the environment. This scenario may, for example, play out when communication is asynchronous with either cognitive or emotional conflicts arising, or in synchronous environments where facial expressions and/or body language cannot convey context information to others.

While communicating with others in order to coordinate activities is biologically primary and so in itself is an activity unlikely to impose a heavy cognitive load, in education contexts the biologically secondary, domain-specific information subjects about which we must communicate and coordinate are highly likely to require the manipulation of a lot of information and carrying out many cognitive activities. Coordinating the acquisition of biologically secondary information can be expected to impose a heavy working memory load. We may need to be taught how to communicate and coordinate carrying out complex tasks in order to optimize transactive activities and construct better knowledge and skill schemas (Zambrano et al. 2018).

### Transactions and instructional design

Because cognitive load theory has largely focused on individual learners performing an individual task, the cognitive load associated with initiating and maintaining communication and coordination – the transaction activities – have not received specific attention. However, collaborative learning environments can only be effectively designed if these activities and their concomitant cognitive load are taken into account. Actors influencing the amount of cognitive load imposed are, for example, the size of the team (i.e., the number of learners per team), the make-up of the team (i.e., the level of expertise of the team members), and the prior collaborative experience of the team members with each other. These factors can be controlled by instructional decisions to promote productive cognitive load for learning.

Situating collaborative learing in a CSCL environment is such an instructional environment that controls the amount of cognitive load that is placed upon the learners. CSCL environments can, for instance, be designed to support group members establish *group awareness*; group members’ knowledge of how the group is functioning and how expertise is divided in the group (cf., Bodemer and Dehler 2011; Engelmann et al. 2010; Engelmann and Hesse 2010, 2011; Janssen et al. 2011; Schreiber and Engelmann 2010). CSCL environments can also be arranged to stimulate students to explicate their claims and arguments by offering *representational guidance* (Schwarz et al. 2003; Janssen et al. 2009). This group awareness and representational guidance reduces group members’ efforts to coordinate their actions, increases group efficiency, and reduces the chance of errors (Gutwin and Greenberg 2004) which in turn reduces unproductive transactive activities and, thus, their cognitive load.

The load incurred/imposed by transactions can be classified as extraneous when the transaction costs incurred negatively impact/are ineffective for learning because they foster errors, conflicts, unnecessary duplication, etc. (Bernard & Lundgren-Cayrol, 2001; Webb & Palincsar, 1996). The extraneous or unproductive cognitive load should be minimised for collaborative learning to be effective. If these costs are not controlled and minimised, the freed-up WM-capacity at the individual and group level could be used for non-essential or non-learning related communication instead of constructing high quality cognitive schemas. All learners need to know their role in the group enterprise. If they do not know how to collaborate or if they are allocated an activity that they cannot fulfil, the act of collaboration may impose an extraneous cognitive load. The advantage of being able to share the cognitive load that a complex task causes could be annulled by too high transaction costs.

In this way, from an instructional perspective, cognitive load theory predicts a better (i.e., more effective) and more efficient collaborative performance in tasks with high complexity (F. Kirschner et al. 2011) where, for instance, groups of learners who have worked as a team in relevant tasks (i.e., the prior collaborative experience principle) are formed. For a math learning problem, each group member received some segments of essential information meant to reduce the cognitive load and promote communication and coordination with each other (i.e., transactive activities). The advantage of having worked as a group in a mathematical task means that the group may have acquired a group schema on how to interact to solve an analog problem (i.e., generalized, collective, domain knowledge, Kalyuga, 2015; Kalyuga & Hanham, 2011). That is, learners may know how to share task essential information, how to perform shared computations for each task step, how to control the amount of time spent in the subtasks, ask for clarifications about the calculations or results obtained, monitor if each member is doing the calculations correctly, make sure to get an appropriate result according to the specific-domain knowledge, and so forth.

These transactive activities impose load as learners need to process both information essential to solve the task as well as unrelated information that can contribute to both individual and group learning. This is the case when two or more members carry out shared calculations, and a third member does not understand how they got to that answer and asks for explanation, thus updating her/his mental calculations by comparing them with the explanation of the peers. Although the cognitive load of these transactive interactions is not intrinsic to the task, they are productive (i.e., are germane to completing the learning task and to the subsequent learning) so that the third group member acquires new knowledge along with a better structure of knowledge about the task. A group that has not had previous collaborative experience in solving a specific domain task would invest more working memory resources in, for example, organization and coordination interactions to carry out the task. Such groups would be expected to have lower performance as their members need to learn to collaborate while attempting to carry out the learning task along with learning from their efforts. A group with previous collaborative experience in solving a specific domain task would be expected to perform better because the resources of its collective work memory are invested in productive transactive activities for learning.

## The Collaborative Learning Context

The collaborative learning context can be seen as the interaction between the learning task, the individual learners, and the team. Each specific collaborative learning situation is influenced by the characteristics of each of these three constituent factors.

### Task Characteristics

A collaborative learning task is a concrete, authentic whole-task learning experience that has to be completed within a given period of time in collaboration with other learners. The task can take many forms, for example, an assignment, a problem that has to be solved, or a project that needs to be carried out. It can also be convergent or divergent and can be well-structured, ill-structured, or even wicked. Whatever the type, *task complexity* is key. Collaboration will occur when the task is complex enough to justify the extra time and effort involved in collaborating with others.

#### Task guidance & support

Carrying out a learning task in any learning situation requires good support and guidance (Van Merriënboer & Kirschner, 2018). This is even more the case in collaborative learning situations as research on this has repeatedly shown that learners typically do not engage in effective collaboration processes without guidance (Weinberger et al. 2007). Guidance is typically process-oriented to help learners systematically approach the learning task guiding them through the phases. In CSCL, collaboration scripts (Fischer et al. 2013) are often used to guide learners’ activities in CSCL settings. Support can be either product-oriented (e.g., worked examples (Kirschner 2002b; Schwaighofer et al. 2017); representations (Suthers 2003, 2006; Van Bruggen et al. 2002) or process-oriented (e.g., assigning roles; Schellens et al. 2007) and is intended to help learners carry out a learning task that could otherwise not be performed without that help. The amount and type of guidance and support offered to learners will affect their ability to carry out the task and thus will also affect the cognitive load experienced by them.

### Learner Characteristics

From a cognitive load theory perspective, the major differences between individuals that have instructional design implications for collaborative learning include the amount of domain-specific knowledge that learners have with respect to the task and the degree of expertise in the mechanics of collaboration.

#### Domain-specific expertise

When teams are composed of learners with a low level of domain-specific knowledge, these novices need to be involved in cognitively demanding search-based problem solving, whereas when they are knowledgeable, this is not the case as the learners can probably deal with the problems using their available knowledge base. Also, when teams are composed of learners with a low level of domain-specific knowledge, there is a greater potential for a larger increase in collective WM than when individuals have high levels of domain-specific knowledge required by the task.

#### Collaboration skills

Besides domain-general collaboration skills which are biologically primary and thus unlikely to be affected by instruction, task-specific collaboration skills can be influenced. These skills relate to team members’ abilities to properly orient themselves to a specific task (Fransen et al. 2011). With respect to transactional activities and their concomitant costs, it is to be expected that the availability of those skills will lower the costs as teams where members have these skills will need to communicate and overtly coordinate their activities less than in teams where these skills have not been acquired. In terms of cognitive load, if learners have not acquired these skills prior to beginning on the collaborative task, the load induced here could be so high as to hinder collaborative learning..

### Team Characteristics

With respect to collaborative learning, four characteristics seem to be important, namely: team size, the roles learners can or must carry out, team composition, and the prior experience of team members working with each other.

#### Team size

The *size* of a team plays a role in how the team members will interact with each other and how effective and efficient the teamwork process will be. In general, the larger the team, the more complex the collaboration process will be (i.e., the more transactive activities that will need to take place) and the greater the risk of social loafing, free riding, and ultimately of the team floundering and failing. With respect to cognitive load, the larger the team, the more transactive activities will be needed to coordinate learner actions and the more communication that will be needed within the team. This will be partially compensated by a lower load resulting from the collective working memory effect if the task is sufficiently complex.

#### Team roles

Roles (e.g., chair, timekeeper, reporter, etc.) promote team cohesion and responsibility (Mudrack & Farrell, 1995; Strijbos, Martens, Jochems, & Broers, 2004). They make clear who has responsibility for what and as such, when roles are either pre-assigned by the instructor or chosen by the learners themselves, they should reduce the coordination activities of the team members. With respect to cognitive load, by reducing coordination activities, roles should reduce the cognitive load incurred by transactive activities.

#### Team composition

The *composition* of a team in terms of the team members’ domain-specific knowledge or expertise also plays a role. Zhang et al. (2016) hypothesised that heterogeneous teams (composed of novice and knowledgeable learners) could be favourable for learners with lower levels of prior knowledge. When teams are homogeneous, novices are involved in cognitively demanding search-based problem solving. When they are knowledgeable learners, homogeneity may be of no benefit since these learners can probably deal with the problems using their available knowledge base. In general, the results confirmed this; however, they also found that when participants have relevant task knowledge, individual learners marginally outperform homogeneous and heterogeneous teams. With respect to cognitive load, if learners have relevant knowledge to carry out a task, communication and coordination activities may be unnecessary or even detrimental to learning. When there is little domain-specific knowledge, the cognitive load incurred by transactions could positively impact learning but where there is a great degree of expertise, and thus where transactions are either unnecessary for or detrimental to learning (Zambrano et al. 2017b), the cognitive load incurred could negatively impact learning.

#### Prior team experience collaborating on similar tasks

Prior experience working on tasks similar in structure to a new learning task allow learners to acquire task-specific collaboration skills associated with higher instructional effectiveness (i.e., performance) and efficiency (i.e., favourable combination of performance and mental effort). With respect to cognitive load, teams in which the members have experience with each other on tasks similar to the learning task will need fewer transactional activities as they know how each other works, what each other knows, and share mental models. As such, the load imposed by these activities will be lower than by non-experienced, ad-hoc teams.

The aspects discussed in this section lead to a number of principles (see Table 2).

**Table 2:** Collaborative Cognitive Load Principles

Principle	Description
Task complexity	Effective collaboration occurs when a task is complex enough to justify the extra time and effort involved in the necessary transactional activities. If a task is not complex enough, unnecessary transactional activities will cause extraneous cognitive load and will, thus be detrimental to learning.
Task guidance & support	When learners face new collaborative situations and environments (e.g., in CSCL), the more guidance and support a task provides for collaborative learning, the lower the extraneous load caused by transactive activities.
Domain expertise	The greater the expertise of team members in the task domain, the lower the extraneous load caused by transactive activities.
Collaboration skills	The availability of collaboration skills of the team members will lower the extraneous load caused by transactive activities.
Team size	The more members that a team working on a learning task, the higher the number of transactive activities, and thus the extraneous load caused by transactive activities.
Team roles	Team roles make clear who has responsibility for what and as such will lower the extraneous load caused by transactive activities.
Team composition	The more heterogeneous the knowledge distribution among team members working on a learning task, the higher the extraneous load caused by transactive activities.
Prior task experience	The more experience team members have coordinating their actions on tasks in general (i.e., they know what to expect from each other in terms of task execution), the lower extraneous load caused by transactive activities.
Prior team experience	The more experience team members have working with each other on a learning task, the lower the extraneous load caused by transactive activities.

## Conclusion

The general framework used by cognitive load theory is directly applicable to collaborative learning but with specific additions to account for collaboration, namely the mutual cognitive interdependence principle (Tomasello and Gonzalez-Cabrera 2017; Tomasello, Melis, Tennie, Wyman, & Herrmann, 2012). The concepts of biologically primary and secondary knowledge from evolutionary educational psychology are relevant to collaborative learning as is the cognitive architecture on which the theory is based. The distinction between intrinsic and extraneous cognitive load is equally relevant to both individual and collaborative learning. All translate directly and easily to collaborative learning. The major additions required when dealing with collaborative learning are the concepts of a collective working memory along with the effects due to the transactive activities associated with the multiple individual working memories that constitute the collective working memory. These additions provide novel hypotheses associated with the effects of differential domain-specific knowledge on collaborative effectiveness and the potential for novel instructional effects for the context of CSCL.

Collaborative Cognitive Load Theory indicates that the possibilities and limitations of collaborating group members, should be taken into account when making informed decisions concerning the design of effective collaborative learning environments. Without this consideration the outcomes of collaborative learning evironments will remain unpredictable and mixed. A teacher informed by Collaborative Cognitive Load Principles who uses collaborative learning as an instructional intervention should first explicitly think about the cognitive properties of his students (e.g., novice or expert) and the effects that the task (e.g., low or high complexity) and group composition (e.g., heterogenous or homogenous) will have on the cognitive processes that will take place. Based upon the learning goal (e.g., learning domain-specific knowledge or interdisciplinary learning) the teacher can make an informed decision that will increase the chances of the learning goals being met. This decision could very well be to not use collaborative learning.

## Footnotes

1 The terms ‘team’ and ‘group’ are used interchangeably in this article.

2 Geary uses the terms ‘knowledge’, ‘skills’, and ‘information’ almost interchangeably in his writings. As such, we also use these terms when discussing biologically primary and secondary aspects of learning.
